# Localization of O₂‑sensing ADO‑RGS pathway components in mouse and human kidneys under normoxia, hypoxia and renal fibrosis

**DOI:** 10.1007/s00424-026-03188-7

**Published:** 2026-06-25

**Authors:** BKM Firmke, A-L Forst, C Daniel, G Schley, KAE Broeker

**Affiliations:** 1 Physiology I, Institute of Physiology, University of Regensburg, Universitätsstraβe 31, 93053 Regensburg, Germany; 2 Medical Cell Biology, Institute of Physiology, University of Regensburg, Regensburg, Germany; 3 Department of Nephropathology, Faculty of Medicine, Friedrich-Alexander-Universität (FAU) Erlangen-Nürnberg and Uniklinikum Erlangen, Erlangen, Germany; 4 Department of Nephrology and Hypertension, Friedrich-Alexander-Universität (FAU) Erlangen-Nürnberg and Uniklinikum Erlangen, Erlangen, Germany

**Keywords:** ADO-RGS-signaling pathway, O_2_-sensing, Hypoxia, Fibrosis, Chronic kidney disease, Spatial transcription

## Abstract

Cellular adaptation to hypoxia is essential for maintaining function and survival. While most hypoxic responss are mediated by hypoxia‑inducible factor signaling, the oxygen‑dependent ADO–RGS (2‑Aminoethanethiol-Dioxygenase—Regulator of G‑Protein Signaling) pathway has recently been implicated in oxygen‑sensitive regulation of G‑protein signaling. The kidneys are particularly vulnerable to hypoxia, a major contributor to chronic kidney disease. Because a systematic characterization of ADO and its RGS substrates across renal cell types is lacking, this study examined their spatial expression patterns in mouse and human kidneys under (patho)physiological conditions. *Ado* and *Rgs4*, *Rgs5*, and *Rgs16* expression was mapped in mouse kidney sections under normoxic, hypoxic, and fibrotic conditions using RNAscope™, complemented by RT‑qPCR. Mouse data were compared with *ADO*-*RGS* expression patterns in human biopsies. *Ado* expression was uniform across renal regions, cell types, and conditions. Besides its baseline presence in vascular cells, *Rgs4* showed strong induction in cortical and outer medullary fibroblasts during anemia. It was also upregulated in fibroblasts and proximal tubules within fibrotic lesions. *Rgs5* was highly expressed in vascular structures and demonstrated hypoxia‑induced upregulation in medullary fibroblasts and *vasa recta*, with moderate induction under fibrotic conditions. Tubular epithelial expression also occurred during fibrosis. *Rgs16* was mostly expressed in *Pdgfrb⁺* interstitial cells in fibrotic kidneys. Human kidney-disease biopsies also displayed distinct *RGS4* and *RGS5* expression patterns. Overall, these findings suggest that while ADO is consistently present, the functional impact of ADO–RGS signaling may be driven by dynamic, cell‑type‑specific regulation of RGS genes during acute and chronic hypoxic stress.

**Graphical Abstract:**

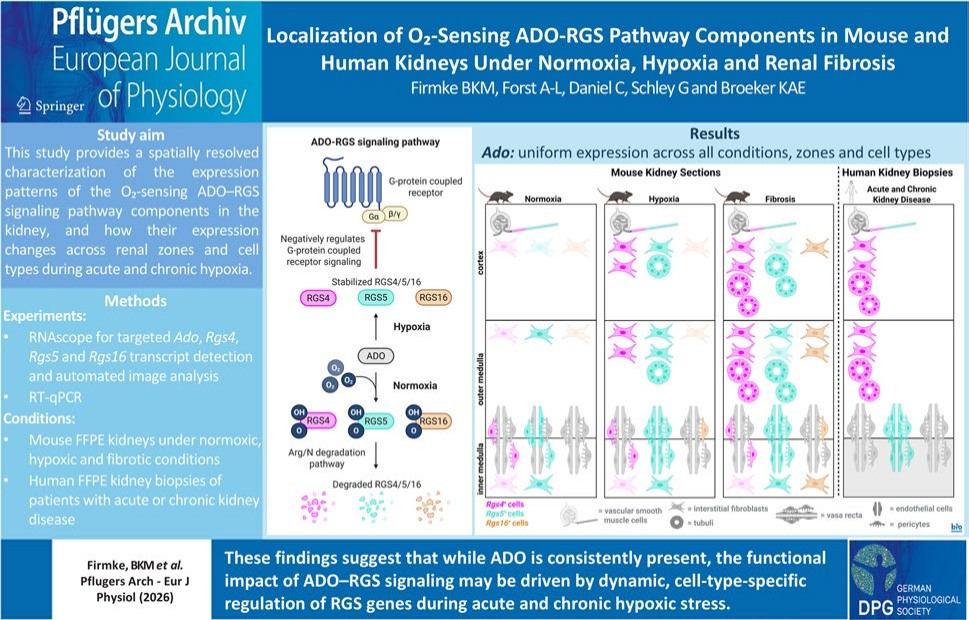

**Supplementary Information:**

The online version contains supplementary material available at 10.1007/s00424-026-03188-7.

## Introduction

Chronic kidney disease (CKD) affects approximately 10% of the global population, and its prevalence has nearly doubled over the past 35 years. CKD is characterized by a progressive decline in renal function, accompanied by interstitial fibrosis and renal anemia. Another hallmark feature of CKD is the development of chronic hypoxia, which results from multiple pathological processes, including the loss of peritubular capillaries, reduced erythropoietin (EPO) production, impaired oxygen diffusion, oxidative stress, and sustained inflammation. Importantly, hypoxia is not only a consequence of CKD; it also plays a central role in initiating and accelerating disease progression. Proximal tubular epithelial cells are particularly susceptible to hypoxic injury due to their exceptionally high energy and oxygen demands [33, 50].

Renal cells distinctly response to hypoxia. During acute hypoxia, interstitial fibroblasts increase EPO synthesis to stimulate erythropoiesis, while tubular epithelial cells shift their energy metabolism from fatty acid oxidation to enhanced glycolysis. During long-term hypoxia this metabolic reprogramming, however, results in the activation of proinflammatory and profibrotic signaling, thereby exacerbating tissue damage. Metabolic reprogramming to increase glycolysis has also been reported for myofibroblasts [17, 33].

Many mechanisms of cellular adaptation to oxygen deficiency are governed by the hypoxia‑inducible signaling pathway, mediated by the hypoxia‑inducible transcription factors (HIF)‑1α and HIF‑2α. Under normoxic conditions, prolyl‑4‑hydroxylases (PHDs) hydroxylate the HIF‑α subunits in an oxygen‑dependent manner, marking them for rapid proteasomal degradation. When oxygen levels decline, hydroxylation is inhibited, allowing HIF-α subunits to evade degradation, accumulate, and translocate to the nucleus, where they activate the transcription of HIF target genes [15, 40].

More recently, an additional conserved oxygen‑sensing mechanism that operates independently of the canonical HIF pathway has been identified: the ADO–RGS pathway (ADO, 2-Aminoethanethiol-Dioxygenase, cysteamine dioxygenase; RGS, Regulator of G‑Protein Signaling) [22, 32]. ADO catalyzes the oxygen‑dependent dioxygenation of N‑terminal cysteine residues on some proteins that contain a Met‑Cys motif following removal of the initiating methionine. This reaction converts the cysteine residue into cysteine sulfinic acid, thereby targeting these proteins for proteasomal degradation via the Cys/Arg branch of the N‑degron pathway [18, 21]. Established ADO substrates include the regulators of G‑protein signaling RGS4, RGS5, and RGS16 and interleukin-32 [7, 21, 24]. RGS4, RGS5, and RGS16 function as GTPase‑activating proteins (GAPs) for the heterotrimeric G‑protein α‑subunits Gα_i_ and Gα_q_, and are therefore involved in the regulation of G‑protein signaling pathways. When oxygen levels decline, ADO no longer oxygenizes these RGS proteins, preventing their degradation and allowing them to accumulate. The stabilized RGS proteins can then exert their GAP activity and attenuate G‑protein‑coupled signaling, thereby contributing to the cellular adaptation to reduced oxygen availability [34]. Because the ADO–RGS system directly modulates G-protein-coupled signaling, whereas the HIF pathway primarily regulates the transcription of target genes, the ADO pathway enables faster and more immediate responses to changes in tissue oxygenation [49].

Given the kidney’s high susceptibility to hypoxia, delineating the expression patterns of ADO and RGS proteins under different conditions may provide important insights into the specific cell types in which the oxygen-sensitive ADO–RGS signaling axis may play a role in healthy and diseased kidneys.

However, little is known about the expression patterns of *ADO*, *RGS4*, *RGS5*, and *RGS16* in healthy, hypoxic, or diseased kidneys. As the spatiotemporal expression of RGS proteins is crucial for their correct functions and interactions with target molecules [45, 54], it is essential to identify the renal cell types in which ADO and the respective RGS proteins are expressed under different (patho)physiological conditions. To address this gap, we systematically analyzed the expression patterns of *Ado*, *Rgs4*, *Rgs5*, and *Rgs16* transcripts in mouse kidneys under normoxic, hypoxic and pathophysiological conditions using multiplex RNAscope™. Additionally, *ADO* and *RGS* gene expression were examined in human kidney biopsies from patients with acute or chronic kidney disease.

## Methods

### Ethical approval

Human kidney tissue was provided by the Department of Nephropathology of the Universitätsklinikum Erlangen, and the use of archival material was approved by the Ethics Committee of the Friedrich-Alexander-Universität (FAU) Erlangen-Nürnberg, waiving the need for retrospective informed consent for the use of archived rest material (Re. No.22–150-D). Human research was performed in accordance with the Declaration of Helsinki.

No new animal experiments were required for the analyses in this study. The tissue samples used were obtained from previously approved animal experiments. Authorization for these experiments was granted by the responsible ethics committee of the Government of Lower Franconia (RUF-55.2.2–2532-2–935; RUF‑55.2.2‑2532‑2‑1754; RUF‑55.2.2‑2532‑2‑2173) and had been carried out in accordance with the Directive 2010/63/EU of the European Parliament and of the Council on the protection of animals used for scientific purposes. Animals had been housed under controlled environmental conditions, including a 12:12 h light–dark cycle, a temperature of 22 ± 2 °C, and a relative humidity of 55 ± 10%. All mice received standard rodent chow (0.6% NaCl; Ssniff Spezialdiäten GmbH, Soest, Germany) and had free access to autoclaved tap water.

### Mouse kidney tissue samples

All mouse tissue samples were from wild-type/control mice with a C57/BL6J background. All mice were euthanized at 18–22 weeks of age for organ collection. This applies to all experimental conditions examined. To induce anemia with hematocrit levels of 25 ± 2%, repeated phlebotomy from the facial vein was performed. Mice were maintained for an additional 4 h after reaching the target hematocrit, after which kidneys were harvested for analysis [10]. For pharmacological stabilization of HIF transcription factors, wild-type mice received the prolyl-4-hydroxylase inhibitor (PHDi) roxadustat via micropipette-guided oral administration. Roxadustat was administered at 50 mg/kg body weight (dissolved in 0.5 M Tris–HCl, pH 9.0), with eight doses delivered at 90-min intervals. Kidneys were collected 90 min after the last PHDi administration. Both experimental groups included male and female mice in comparable numbers [10].

Adenine-induced nephropathy (AN) was generated in adult male mice by providing a 0.2% adenine-containing diet (Altromin Spezialfutter GmbH & Co. KG, Lage, Germany) for 3 weeks. Immediately after completion of the diet, kidneys were collected for analysis. Unilateral ureteral obstruction (UUO) was performed under inhalation anesthesia in mice of both sexes. Through a small abdominal incision, the right ureter was ligated close to the kidney. Five days after ligation, animals were euthanized and either perfused for RNAscope™ analysis or kidneys were harvested for mRNA quantification [12].

### Human kidney tissue specimens

Formalin-fixed paraffin-embedded (FFPE) kidney biopsies from patients with acute and chronic kidney disease were selected to investigate hypoxia-induced gene expression in humans. For acute kidney disease we used biopsies from renal transplants taken before transplantation experiencing hypoxia due to lacking oxygen supply after explantation resulting in mild or severe tubular injury (Table 1). In addition, two biopsies were included from patients with progressed IgA nephropathy (IgAN), showing a high degree of global glomerulosclerosis and interstitial fibrosis/tubular atrophy (IFTA). For these patients, severe fibrosis indicates chronic oxygen deprivation in the kidneys. One biopsy from a patient with IgAN but absent fibrosis served as control (Table 1).

**Table 1:** Patient characteristics

Biopsy sample	Gender [m/f]	Age [y]	Injury grading	Glomeruli with global glomerulosclerosis [%]	Acute tubular injury [score 0–4]	IFTA [%]
0-biopsy	nk	nk	mild	0	2	0
0-biopsy	nk	nk	severe	0	4	0
0-biopsy	nk	nk	severe	18	4	0
IgAN	f	44	mild	0	0	0
IgAN	m	74	progressed	75	0	26–50
IgAN	f	33	progressed	71	0	51–75

*nk* not known.

### Organ removal and tissue preparation

For kidney removal, mice were anesthetized with ketamine/xylazine (ketamine 100 mg/kg body weight; xylazine 10 mg/kg body weight, intraperitoneally) and were killed by exsanguination or cervical dislocation. The left kidneys were removed and flash-frozen in liquid nitrogen for mRNA quantification, while the right kidneys were perfusion-fixed for tissue analysis. In UUO animals, both kidneys were either perfusion-fixed or flash-frozen for RNA analyses.

For RNAscope™ tissue preparation, kidneys were perfused with 30 ml sterile phosphate-buffered saline (PBS; pH 7.4), followed by 40 ml of 10% neutral-buffered formalin (pH 7.0). After dehydration through a graded ethanol–isopropanol series, kidneys were embedded in paraffin and sectioned at 5 µm thickness.

### Quantification of renal mRNA expression levels by RT-PCR

RNA was isolated using TRIzol according to the standard guanidinium thiocyanate-phenol–chloroform extraction protocol [5]. Subsequently, 1 µg of total RNA was reverse-transcribed into cDNA using oligo(dT) primers and Moloney murine leukemia virus reverse transcriptase (Thermo Fisher Scientific, Waltham, MA, USA).

Renal mRNA expression levels were quantified by real-time qPCR using a LightCycler 96 instrument and the SYBR Green I Master Kit (Roche Diagnostics, Mannheim, Germany). Ribosomal protein L32 (Rpl32) served as the housekeeping gene for normalization. Primer sequences (Eurofins, Munich, Germany) are provided in Table 2.

**Table 2:** RT-qPCR primer sequences

Genes	Sequence (5´to 3´), fwd	Sequence (5´to 3´), rev	Product size (bp)
*Rpl32*	TTAAGCGAAACTGGCGGAAAC	TTGTTGCTCCCATAACCGATG	100
*Ado*	CTGGCAGGGAAACAAACTGC	TAGCAGCCATGGTGGAAGTG	249
*Rgs4*	AATAGAAACCACCGCGGCTC	GAAAGCTGCCAGTCCACATT	292
*Rgs5*	CCCCATCAAAATGGCGGAGA	TCTGGGCCAAGTCAAAGCTG	152
*Rgs16*	CCATGCCTTCCTAAAGACGGA	GTACTCGTCAAAGATGTGGTGAG	127

Abbreviations: *Rpl32* ribosomal protein L32, *Ado* cysteamine dioxygenase, *Rgs* Regulator of G-protein signaling.

### In-situ hybridization using RNAscope™ technology

*In-situ* hybridization was performed using the RNAscope™ Multiplex Fluorescent v2 Kit (Advanced Cell Diagnostics, Hayward, CA, USA) according to the manufacturer’s instructions [51]. Target mRNAs were hybridized using the probes listed in Table 3 and visualized with the fluorophores TSA Vivid 570, TSA Vivid 650 (Bio-Techne GmbH, Wiesbaden, Germany) and Opal 780 (Akoya Biosciences, Marlborough, MA, USA). Nuclei were counterstained with 4′,6-diamidino-2-phenylindole (DAPI) provided with the kit. After staining, sections were mounted with ProLong Gold Antifade Mountant (Thermo Fisher Scientific, Waltham, MA, USA) and stored at 4 °C in the dark until imaging. Positive and negative control probes were included in each experiment.

**Table 3:** RNAscope™ probes

RNAscope™- Probe	Cat No	RNAscope™- Probe	Cat No
Mm-*Adgre-1*	460651	Mm-*Pdgfrb*-C2	411381-C2
Mm-*Adgre-1*-C2	460651-C2	Mm-*Pdgfrb*-C3	411381-C3
Mm-*Adm*	493601	Mm-*Pecam1*	316721
Mm-*Ado*	570001	Mm-*Pecam1*-C2	316721-C2
Mm-*Ado*-C4	570001-C4	Mm-*Rgs4*	467461
Mm-*Aqp2*-C3	452411-C3	Mm-*Rgs4*-C3	467461-C3
Mm-*Calb1*	428431	Mm-*Rgs5*	430181
Mm-*Cdh16*	582781	Mm-*Rgs5*-C2	430181-C2
Mm-*Clcnka*	536031	Mm-*Rgs16*-C4	539201-C4
Mm-*Col1a1*-C2	319371-C2	Mm-*Slc12a1*-C2	476841-C2
Mm-*Egln3*	434931	Hs-*ACTA2-O1*-C2	444771-C2
Mm-*Epo*	315501	Hs-*ADO*-C3	1696861-C3
Mm-*Epo*-C2	315501-C2	Hs-*CDH16*-C2	1187911-C2
Mm-*Epo*-C3	315501-C3	Hs-*HAVCR1-O1*-C2	538081-C2
Mm-*Havcr1*	472551	Hs-*PDGFRB*	548991
Mm-*Havcr1*-C4	472551-C4	Hs-*PDGFRB*-C2	548991-C2
Mm*-Lcn2*	313971	Hs-*RGS4*-C3	1087221-C3
Mm-*Lrp2*-C2	425881-C2	Hs-*RGS5*-C4	533421-C4
Mm-*Myh11*	316101	Hs-*RGS16-No-XMm*-C4	1059871-C4
Mm-*Panx1*	316321	Negative Control Probe	320751
Mm-*Pdgfrb*	411381	Positive Control Probe	321651

Abbreviations: *Adgre-1* adhesion G protein-coupled receptor E1 of macrophages, *Adm* adrenomedullin, *Ado* cysteamin-dioxygenase, *Aqp2* aquaporin 2, *Calb1* Calbindin 1, *Cdh16* cadherin 16, *Clcnka* chloride channel Ka, *Col1a1* collagen type I alpha chain, *Egln3* Egl nine Homolog 3 (PHD3, Prolylhydroxylase 3), *Epo* erythropoietin, *Havcr1* hepatitis A virus cellular receptor 1 (Kim1, kidney injury molecule-1), *Lcn2* Lipocalin 2,* Lrp2* Lipoprotein receptor-related Protein 2 (Megalin), *Myh11* myosin heavy chain 11, *Panx1* Pannexin1, *Pdgfrb* Platelet-derived growth factor receptor beta, *Pecam1* platelet-endothelial cell adhesion molecule-1, *Rgs* regulator of G-protein signaling, *Slc12a1* solute carrier family 12 member 1 (NKCC2, Na–K-Cl symporter), *ACTA2* actin alpha 2.

### Detection of HIF-2α stabilization

Immunohistochemical staining for HIF-2α stabilization was performed as previously described [10]. Briefly, kidneys were perfused with 3% paraformaldehyde, immediately dehydrated using an ascending methanol–isopropanol series, and embedded in paraffin. For tissue permeabilization, 5 µm-thick sections were boiled in Target Retrieval Solution (Agilent Technologies, Waldbronn, Germany). Sections were blocked using an avidin solution (Avidin/Biotin Blocking Kit; Vector Laboratories, Newark, CA, USA), followed by incubation with 3% hydrogen peroxide, and subsequently blocked with Serum-Free Protein Block (Agilent Technologies). The sections were then incubated overnight at 4 °C with a primary antibody against HIF-2α (polyclonal rabbit anti-HIF-2α, 1:5000 dilution; NB100-122; Bio-Techne) diluted in 1% BSA/PBS. After washing with PBS, sections were incubated with a donkey anti-rabbit HRP-conjugated secondary antibody (1:500 dilution; CS7074; Cell Signaling Technology, Danvers, MA, USA) for 45 min at room temperature. Signal amplification was performed using the TSA Plus Biotin Kit (1:100 dilution, 15 min; Akoya Biosciences), followed by incubation with streptavidin-HRP (Abcam, Cambridge, UK) for 30 min. Signal detection was carried out using freshly prepared 3,3′-diaminobenzidine (DAB) solution (DAB Peroxidase Substrate Kit, Vector Laboratories). Sections were mounted using Dako Glycergel (Agilent Technologies).

### Microscopy

RNAscope™ images were acquired using a Zeiss Axio Observer.Z1 microscope (Zeiss, Jena, Germany) equipped with an Axiocam 506 Mono fluorescence camera. Imaging was performed with a Plan-Apochromat 20 ×/0.8 objective and illuminated using the Colibri 7 LED light source. Fluorescence detection employed the following Zeiss filter sets: 43-Cy3 (EX BP 545/25; EM BP 605/70), 50-Cy5 shift-free (EX BP 640/30; EM BP 690/50), 96 HE BFP (EX BP 390/40; EM BP 450/40), and 115-Cy7 (EX BP 710/87; EM BP 814/91).

Low-magnification overview images of transverse kidney sections were generated by stitching together tiled images. For high-resolution fluorescence analysis, 10–12 optical sections were captured as z-stacks using the Apotome.2 structured illumination module. These stacks were subsequently deconvolved and combined using a maximum-intensity projection.

### Image Analysis

ImageJ software was used to demonstrate the distribution of cells exhibiting a distinct marker combination per kidney section using the “cell counter” tool.

Automated image analysis was carried out using Intellesis (Zeiss) to evaluate co‑expression patterns at the single‑cell level. Prior to quantification, kidney sections were manually divided into cortex and medulla based on established histological criteria. For each fluorescence channel, threshold settings were adjusted to capture all detectable signals across the full dynamic range of the imaging system, up to the maximum measurable intensity of 16,384. Cell nuclei identified by DAPI staining served as anchors for generating zones of interest (ZOIs). A defined radius was applied around each nucleus to approximate the cellular domain, allowing transcripts located within this radius to be assigned to the corresponding cell. Nuclear segmentation and watershed processing were applied to ensure clear separation between adjacent nuclei. Segmentation of target and marker channels was based on global thresholding with a tolerance of 3% and a neighborhood value of 1%. The proportion of target/marker‑positive cells was calculated relative to the number of marker‑positive cells to enable normalization across samples. No restrictions on object size were applied to ensure that all RNAscope™ signals were included in the analysis.

Area‑based measurements were performed to quantify expression of the transcripts across defined kidney regions, including cortex, outer stripe of the outer medulla, inner stripe of the outer medulla, and inner medulla. Automated segmentation was conducted in Intellesis (Zeiss), using background subtraction without any additional smoothing or sharpening steps. Histogram settings for all target channels were adjusted to capture the complete spectrum of RNAscope™ signal intensities up to the maximum measurable value (16,384). Segmentation used 0% tolerance and a neighborhood value of 0%. For DAPI, segmentation employed a tolerance of 3%, and hole filling was activated to ensure accurate nuclear delineation. For each kidney region, the area positive for target signals was quantified and normalized to the DAPI‑positive nuclear area. No signal size thresholds were used in order to include low‑abundance transcripts.

Across all experimental groups, representative kidney overviews of at least 5 different animals per condition were processed and evaluated using this analysis pipeline.

#### Statistical analyses

Data were analyzed using GraphPad Prism 10.6.1 (GraphPad Software Inc., San Diego, CA, USA). Results are presented as mean ± SD. Statistical significance was assessed using one-way ANOVA or Welch ANOVA followed by Dunnett’s post-hoc correction. A p-value ≤ 0.05 was considered statistically significant. Exact p-values and group sizes are reported in the Results section.

#### Localization of ADO-RGS-signaling pathway components in the healthy mouse kidney under normoxic conditions

To systematically determine the expression patterns of *Ado* and its identified substrates *Rgs4*, *Rgs5*, and *Rgs16*, we performed multiplex RNAscope™ assays using target‑specific mRNA probes in combination with various cell type–specific markers. Platelet‑derived growth factor receptor‑β (*Pdgfrb*) mRNA expression was used to identify interstitial fibroblasts/pericytes and intraglomerular mesangial cells; cadherin‑16 (*Cdh16*) served as a marker for tubular epithelial cells; myosin heavy chain 11 (*Myh11*) for vascular smooth muscle cells (VSMCs); and platelet and endothelial cell adhesion molecule 1 (*Pecam1*) for endothelial cells.

**Fig. 1:**
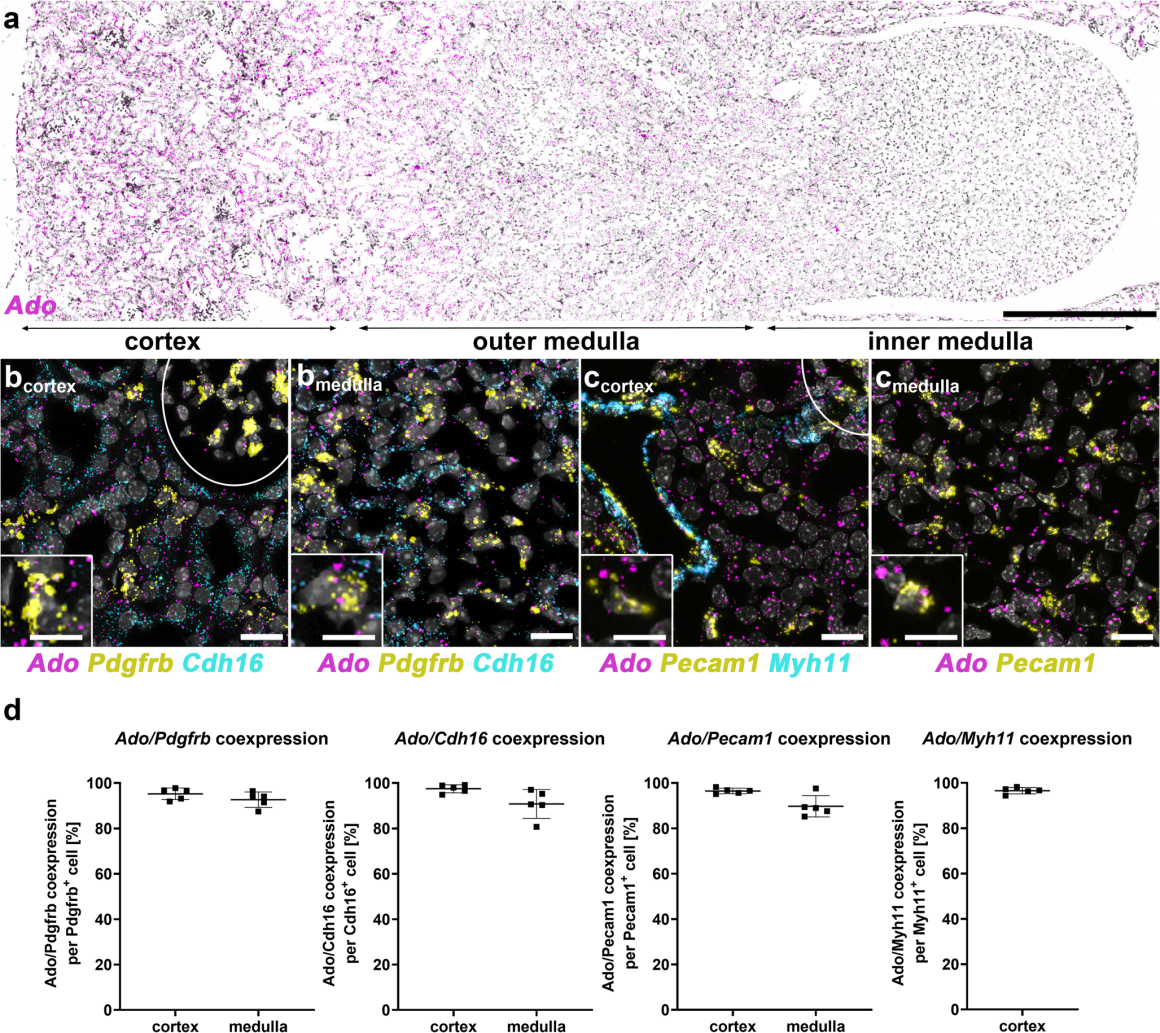
*Ado* expression pattern and colocalization of *Ado* mRNA with various cell markers in normoxic kidneys of wild-type mice. (**a**) Detail from a kidney overview showing the zonal distribution of *Ado* mRNA expression (magenta) using RNAscope™. Nuclei are counterstained with DAPI (grey). Scale bar: 500 µm. (**b**) Details from the cortex and the outer medulla of a normoxic kidney section showing a triple RNAscope™ for *Ado* (magenta) mRNA, the interstitial fibroblast marker *Pdgfrb* (yellow) mRNA and the tubular marker *Cdh16* (cyan) mRNA. Glomerulus is indicated by a white circle. Nuclei are counterstained with DAPI (grey). Scale bars: 20 µm. The square in the lower left corner shows the co-expression of *Ado* and *Pdgfrb* transcripts at higher magnification. Scale bars: 10 µm. (**c**) Details from the cortex and the outer medulla of a normoxic kidney section showing a triple RNAscope™ for *Ado* (magenta) mRNA, the endothelial cell marker *Pecam1* (yellow) mRNA and the VSMC marker *Myh11* (cyan) mRNA. Glomerulus is indicated by a white circle. Nuclei are counterstained with DAPI (grey). Scale bars: 20 µm. The square in the lower left corner shows the co-expression of *Ado* and *Pecam1* transcripts at higher magnification. Scale bars: 10 µm. (**d**) Automated co-expression analysis showing the proportion of *Pdgfrb*^+^, *Cdh16*^+^, *Pecam1*^+^, and *Myh11*^+^ cells that co-expressed *Ado* mRNA. As there are no arteries or arterioles in the medulla, only cortical *Ado*/*Myh11* co-expression is shown. Data are presented as mean ± SD from n = 5 mice per analysis

In tissue sections from normoxic, healthy kidneys of wild-type mice, *Ado* expression appeared relatively uniform and widespread across all renal zones (Fig. 1a). Nearly all *Cdh16*⁺ tubular epithelial cells, as well as *Pdgfrb*⁺ interstitial fibroblasts and intraglomerular mesangial cells, co‑expressed *Ado* mRNA. In addition, most VSMCs and endothelial cells were also positive for *Ado* transcripts (Fig. 1b/c). Automated co-expression analyses confirmed the overlap between *Ado* signals and the respective cell type–specific markers. For each *Ado*/marker combination, the codetection ratio ranged from 90 to 96%, indicating highly consistent and cell type-independent *Ado* expression (Fig. 1d).

**Fig. 2:**
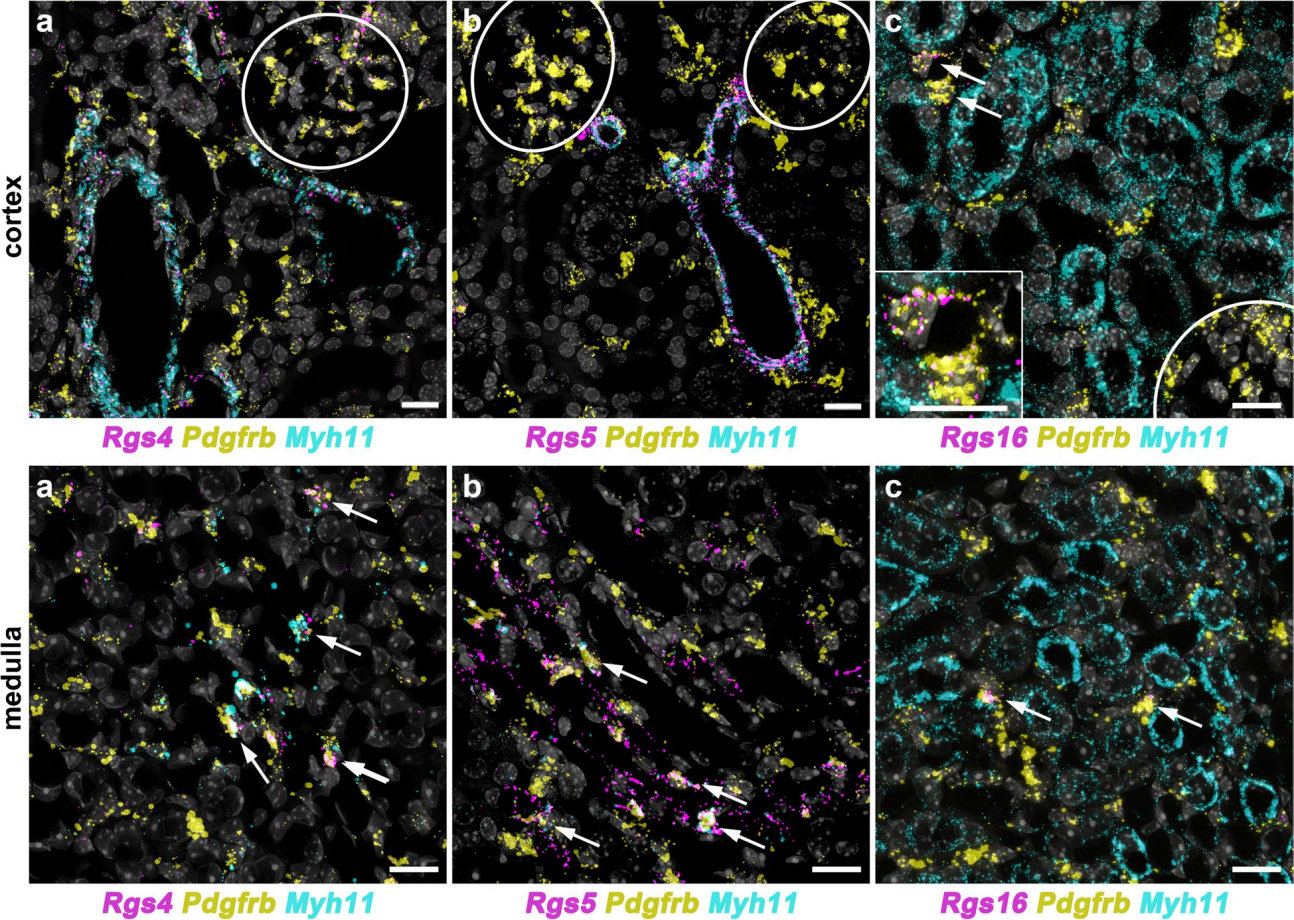
Expression patterns of *Rgs4*, *Rgs5* and *Rgs16* mRNA in the kidneys of wild-type mice under normoxic conditions. (**a**) Details from the cortex and the medulla showing a triple RNAscope™ for *Rgs4* (magenta), *Pdgfrb* (yellow) and *Myh11* (cyan) mRNA. Circle indicates glomerulus. Arrows highlight *Rgs4* positive contractile pericytes (*Pdgfrb/My11*^+^) along *vasa recta*. Scale bars: 20 µm. (**b**) Details from the cortex and the medulla showing a triple RNAscope™ for *Rgs5* (magenta), *Pdgfrb* (yellow) and *Myh11* (cyan) mRNA. Circles indicate glomeruli. Arrows highlight *Rgs5* positive contractile pericytes (*Pdgfrb/Myh11*^+^) along *vasa recta*. Scale bars: 20 µm. (**c**) Details from the cortex and the medulla showing a triple RNAscope™ for *Rgs16* (magenta), *Pdgfrb* (yellow) and *Cdh16* (cyan) mRNA. Circle indicates glomerulus. Arrows highlight *Rgs16/Pdgfrb* coexpressing interstitial cells. Scale bars: 20 µm. The square in the lower left corner shows the coexpression of *Rgs16* and *Pdgfrb* transcripts at higher magnification. Scale bar: 10 µm. In all images nuclei are counterstained with DAPI (grey)

The expression patterns of *Rgs4* and *Rgs5* in normoxic kidneys were largely overlapping. Both *Rgs* isoforms were detected in *Myh11*^+^ VSMCs of cortical arteries and arterioles, as well as in *Pdgfrb*^+^/*Myh11*^+^ contractile pericytes along the medullary *vasa recta*. On a per-cell basis, *Rgs5* mRNA expression was markedly stronger than *Rgs4* expression in these cells (Fig. 2a/b). In contrast to *Rgs4*, weak *Rgs5* mRNA expression was also detected in some *Pecam1*^+^ endothelial cells along the *vasa recta* (Supplemental Fig. 1a).

Additionally, *Rgs4* transcripts were detected in some intraglomerular *Pdgfrb*^+^ mesangial cells, and occasionally in interstitial fibroblasts of the cortex (Fig. 2a), while *Rgs5* could be detected in some interstitial *Pdgfrb*^+^ cells throughout the medulla. *Rgs16* expression was observed only sporadically and without a discernible pattern in *Pdgfrb*^+^ interstitial cells in both the cortex and medulla (Fig. 2c).

Tubular and endothelial cells (except *Rgs5* in *vasa recta*) were negative for *Rgs4*, *Rgs5* and *Rgs16* in normoxic wild-type mouse kidneys.

#### Hypoxemia-induced changes in the expression of ADO–RGS signaling pathway components in wild-type mouse kidneys

Since ADO has been reported to regulate the stability of RGS4, RGS5, and RGS16 in an oxygen-dependent manner, ADO-RGS-signaling is expected to become active when oxygen availability declines [22, 32]. We therefore examined the mRNA expression patterns of *Ado* and *Rgs4*, *Rgs5*, and *Rgs16* under hypoxemic conditions by analyzing kidney sections from wild-type mice rendered anemic by phlebotomy, which reduced hematocrit levels to approximately 25%.

Because we previously observed HIF-2α-dependent induction of *Rgs4* mRNA in Epo-producing fibroblasts [4], we assessed whether *Ado*, *Rgs5*, and *Rgs16* transcription is regulated by HIF signaling. To this end, kidney sections from wild-type mice treated with the prolyl-4-hydroxylase inhibitor (PHDi) roxadustat were included. PHDi administration enables pharmacological stabilization of HIF-1α and HIF-2α under normoxic conditions, and the applied regimen stabilized HIF throughout all renal zones [10]. This is relevant because PHD activity—like ADO enzymatic activity—decreases as O_2_ levels fall [6]. Notably, PHD inhibitors do not interfere with ADO activity because the enzymes employ distinct catalytic mechanisms [49]. Thus, this approach allowed us to distinguish O_2_-dependent from HIF-dependent changes in *Ado*, *Rgs4*, *Rgs5*, and *Rgs16* expression.

**Fig. 3:**
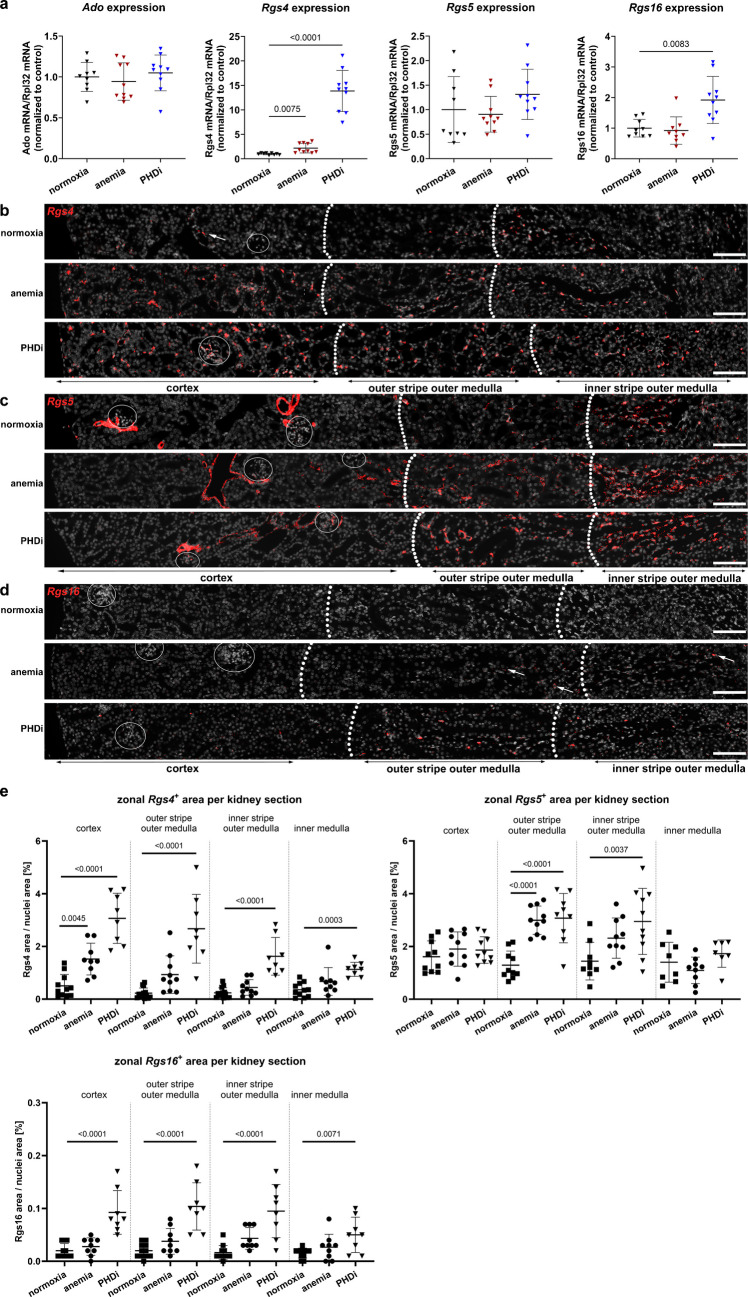
Renal expression levels and patterns of *Ado*, *Rgs4*, *Rgs5* and *Rgs16* mRNA in anemic and PHDi-treated wild-type mice as well as zonal distribution of *Rgs4*, *Rgs5 and Rgs16* transcripts. (**a**) RT-qPCR showing changes in the renal expression of *Ado* and the *Rgs* isoforms under anemic and PHDi-treated conditions compared to normoxic conditions. Statistical significance was determined using Welch ANOVA with Dunnett´s T3 multiple comparisons test. P values are stated above the respective lines. Data represent mean ± SD of n ≥ 9 mice per condition. (**b**) Details from kidney sections under normoxic, anemic and PHDi-treated conditions showing the zonal distribution of *Rgs4* (red) mRNA expression using RNAscope™. Arrow highlights *Rgs4* transcripts in cortical interstitial fibroblasts. Circles indicate glomeruli. Dotted white lines indicate the zonal borders. Nuclei are counterstained with DAPI (grey). Scale bars: 500 µm. (**c**) Details from kidney sections under normoxic, anemic and PHDi-treated conditions showing the zonal distribution of *Rgs5* (red) mRNA expression using RNAscope™. Circles indicate glomeruli. Dotted white lines indicate the zonal borders. Nuclei are counterstained with DAPI (grey). Scale bars: 500 µm. (**d**) Details from kidney sections under normoxic, anemic and PHDi-treated conditions showing the zonal distribution of *Rgs16* (red) mRNA expression using RNAscope™. Arrows highlight *Rgs16* transcripts in interstitial fibroblasts. Circles indicate glomeruli. Dotted white lines indicate the zonal borders. Nuclei are counterstained with DAPI (grey). Scale bars: 500 µm. (**e**) Automated image analysis shows the *Rgs4* (top left), *Rgs5* (top right) and *Rgs16* (bottom left) positive area per indicated kidney zone relative to the nuclei area under normoxic and anemic conditions and after PHDi administration. Statistical significance between conditions within each zone was evaluated using a one-way ANOVA with Dunnett’s post hoc multiple-comparisons test. P values are stated above the respective lines. Data represent mean ± SD of n ≥ 8 mice per condition

We quantified mRNA levels under normoxic, anemic, and PHDi‑treated conditions. RT-qPCR revealed no changes in *Ado* mRNA abundance in anemic or PHDi-treated kidneys compared to normoxic kidneys. In contrast, *Rgs4* mRNA expression was significantly increased in both anemic and PHDi-treated kidneys. *Rgs16* mRNA expression was significantly upregulated only in PHDi-treated kidneys, whereas *Rgs5* mRNA levels remained unchanged under both conditions (Fig. 3a).

RNAscope™ analyses of kidney sections from anemic and PHDi‑treated mice confirmed that *Ado* transcript distribution and abundance were unchanged across conditions (Supplemental Fig. 2). Consistent with RT‑qPCR results, zonal *Rgs4* mRNA expression was increased: while only a few cortical fibroblasts expressed *Rgs4* in normoxic kidneys, anemic kidneys displayed a marked rise in the number of *Rgs4*^+^ fibroblasts in the cortex and the outer stripe of the outer medulla. PHDi treatment further increased the number of *Rgs4*-expressing fibroblasts not only in the cortex but also broadly in the outer and inner medulla (Fig. 3b/e). These differences in interstitial *Rgs4* induction are likely attributable to differences in the extent of HIF-2α stabilization observed under anemia versus PHD inhibition. Consistent with our previous findings [10], HIF-2α stabilization in the kidneys of anemic mice was restricted to fibroblasts of the cortex and the outer stripe of the outer medulla. In contrast, following PHDi-treatment, a greater number of HIF-2α–positive interstitial cells were detected not only in the cortex but also in the corticomedullary region. Moreover, HIF-2α stabilization was observed in interstitial fibroblasts within the inner stripe of the outer medulla and in the inner medulla (Supplemental Fig. 3). Additionally, PHD inhibition led to HIF-2α stabilization in intraglomerular mesangial cells, endothelial cells, and vascular smooth muscle cells.

RNAscope™ analysis also revealed induction of *Rgs5* mRNA expression in interstitial fibroblasts under anemic and PHDi‑treated conditions, predominantly in the deep cortex and outer stripe of the outer medulla. Moreover, *Rgs5* expression increased in medullary pericytes and endothelial cells along the *vasa recta* (Fig. 3, Supplemental Fig. 1b/c), while expression in VSMCs remained unchanged (Fig. 3c/e). Notably, some intercalated cells in the collecting duct started to express *Rgs5* mRNA (Supplemental Fig. 1d).

**Fig. 4:**
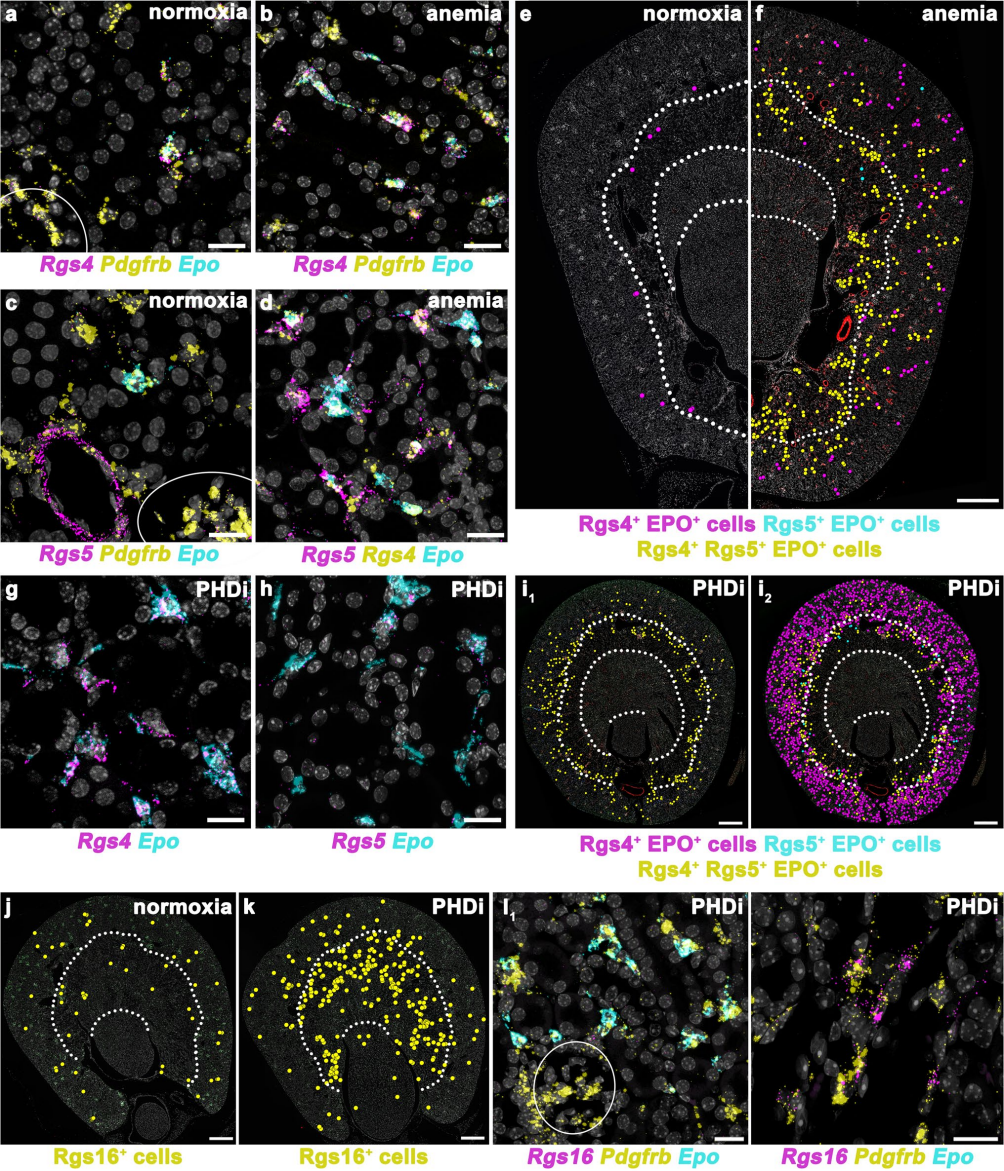
Co-expression of *Epo* with *Rgs4*, *Rgs5*, and *Rgs16* mRNA in interstitial fibroblasts of anemic and PHDi-treated wild-type mice. (**a**/**b**) Cortical details of kidney sections from normoxic (**a**) and anemic (**b**) mice showing RNAscope™ signals for *Rgs4* (magenta), *Pdgfrb* (yellow), and *Epo* mRNA (cyan). Circle highlights glomerulus. Scale bars: 20 µm. (**c**) Cortical detail of a normoxic kidney section showing RNAscope™ signals for *Rgs5* (magenta), *Pdgfrb* (yellow), and *Epo* mRNA (cyan). Circle highlights glomerulus. Scale bar: 20 µm. (**d**) Corticomedullary detail of a kidney section under anemic conditions illustrating the co-expression of *Rgs5* (magenta), *Rgs4* (yellow), and *Epo* mRNA (cyan). Scale bar: 20 µm. (**e**/**f**) Distribution of Rgs4^+^ Epo^+^ cells (magenta dots), Rgs5^+^ Epo^+^ cells (cyan dots) and Rgs4^+^ Rgs5^+^ Epo^+^ cells (yellow dots) on kidney sections under normoxic (**e**) and anemic (**f**) conditions. Dotted white lines indicate zonal borders. Scale bars: 500 μm. (**g**/**h**) Details from the outer stripe of the outer medulla from PHDi‑treated kidneys showing co‑localization of *Rgs4* (**g**) or *Rgs5* (**h**) transcripts (magenta) with *Epo* mRNA (cyan) using RNAscope™. Scale bars: 20 µm. (**i**) Distribution of Rgs4⁺ Epo⁺ (magenta dots), Rgs5⁺ Epo⁺ (cyan dots), and Rgs4⁺ Rgs5⁺ Epo⁺ cells (yellow dots) in PHDi‑treated kidney sections. (i_1_) highlights exclusively Rgs4⁺ Rgs5⁺ Epo⁺ cells (yellow). Zonal regions are outlined with dotted white lines. Scale bar: 500 µm. (**j**/**k**) Distribution of Rgs16^+^ Pdgfrb^+^ cells (yellow dots) on kidney sections under normoxic (**j**) and PHDi-treated (**k**) conditions. Dotted white lines indicate the zonal borders. Scale bars: 500 μm. (**l**) Details from kidney sections under PHDi-treated conditions showing the co-expression of *Rgs16* (magenta), *Pdgfrb* (yellow) and *Epo* (cyan) mRNA using RNAscope™. Circle highlights glomerulus. Scale bars: 20 µm. In all images nuclei were counterstained with DAPI (grey)

No significant upregulation in *Rgs16* mRNA expression was observed under anemic conditions, whereas PHDi treatment increased the *Rgs16*^+^ area and the number of *Rgs16*/*Pdgfrb*‑positive interstitial cells in all zones, but particularly in the outer medulla (Fig. 3d/e, Fig. 4). However, compared with *Rgs4* and *Rgs5*, the *Rgs16*⁺ area per kidney section was consistently smaller under all conditions.

#### Co-expression of Epo with ADO–RGS Signaling pathway components in interstitial fibroblasts

As interstitial *Pdgfrb*^+^ cells are known to produce Epo in a HIF-2α dependent manner [36], coexpression of *Rgs4*, *Rgs5*, and *Rgs16* transcripts with *Epo* mRNA was examined in normoxic, anemic, and PHDi-treated kidneys.

In line with our previous observations, Epo production was restricted to very few fibroblasts located along the corticomedullary border under normoxic conditions (approximately 15 cells per kidney cross-section) (Fig. 4e) [4]. During anemia, the number of Epo-producing fibroblasts increased markedly, with most Epo-positive cells found in the deep cortex and the outer stripe of the outer medulla. Epo-cell density gradually decreased toward the superficial cortex (Fig. 4f). In contrast, following PHDi treatment, nearly all fibroblasts in the cortex and in the outer stripe of the outer medulla expressed *Epo* mRNA (Fig. 4i_2_) [10].

In all three conditions, Epo-producing *Pdgfrb*⁺ fibroblasts in the cortex and outer medulla consistently coexpressed *Rgs4* mRNA (Fig. 4). In contrast, the coexpression pattern of *Rgs5* and *Epo* differed markedly: under normoxic conditions, the few *Epo*-expressing cells were negative for *Rgs5* transcripts (Fig. 4c), whereas under anemic conditions – when *Rgs5* expression expanded to numerous additional *Pdgfrb*⁺ interstitial fibroblasts in the deep cortex and outer stripe of the outer medulla – most *Epo*-producing cells in these regions showed strong coexpression of *Rgs5* (and *Rgs4*) (Fig. 4d). Most *Epo*⁺ fibroblasts in the more superficial regions of the cortex, expressed only *Rgs4* but not *Rgs5* mRNA (Fig. 4f). Additionally, some interstitial fibroblasts expressing both *Rgs4* and *Rgs5* but lacking *Epo* expression were detected in the cortex and outer medulla (Fig. 4d).

Notably, in PHDi‑treated kidneys, *Rgs4*/*Rgs5*/*Epo* triple‑positive fibroblasts were detected in a pattern similar to that observed under anemic conditions. However, the majority of *Epo*⁺ fibroblasts throughout the cortex were *Rgs5*‑negative, despite all of them expressing *Rgs4*, as described above (Fig. 4 g-i). For *Rgs16*, no coexpression with *Epo* was observed under either anemic conditions or following PHD inhibition (Fig. 4j-l).

#### Expression patterns of ADO-RGS-signaling pathway components in mouse kidneys under pathophysiologic conditions

Tissue hypoxia is both a driver of kidney injury and a hallmark of progressive fibrosis, for example due to capillary rarefaction [33, 50]. Therefore, we analyzed the expression of *Ado* as well as *Rgs4*, *Rgs5*, and *Rgs16* in two well-established models of kidney disease – adenine‑induced nephropathy (AN) and unilateral ureteral obstruction (UUO). Target mRNA expression was quantified in whole‑kidney lysates, and spatial expression patterns were examined by RNAscope™.

**Fig. 5:**
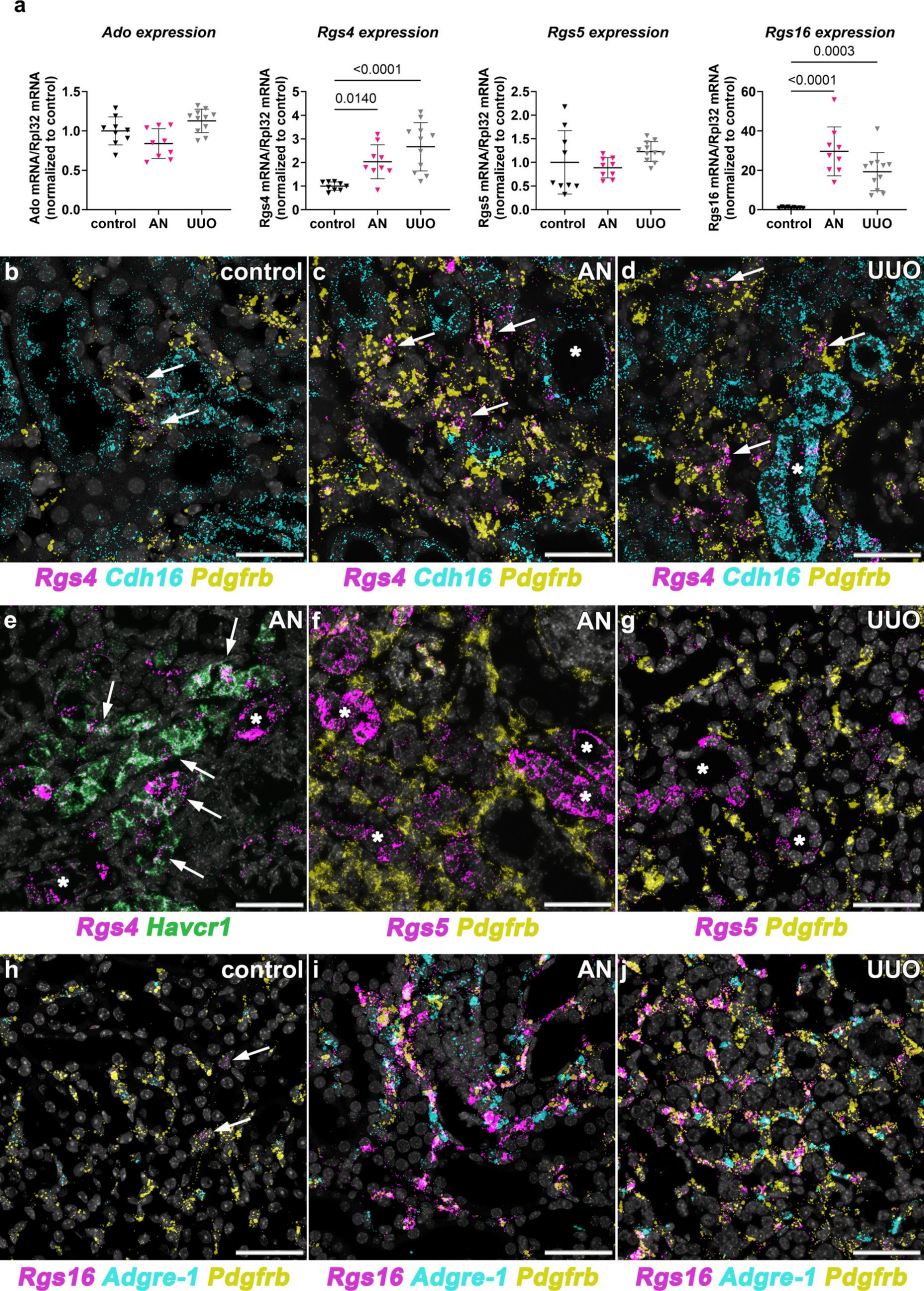
Renal expression levels and patterns of *Ado*, *Rgs4*, *Rgs5* and *Rgs16* mRNA in control, AN and UUO mice. (**a**) RT-qPCR showing changes in the renal expression of *Ado* and the *Rgs* isoforms under AN and UUO compared to respective control mice. Statistical significance was determined using one-way ANOVA with Dunnett´s multiple comparisons test. P values are stated above the respective lines. Data represent mean ± SD of n ≥ 9 mice per condition. (**b**-**d**) Corticomedullary details of kidney sections from control, AN and UUO mice showing triple RNAscope™ for *Rgs4* (magenta), *Cadherin16* (cyan) and *Pdgfrb* mRNA (yellow). Arrows highlight *Rgs4*/*Pdgfrb*-coexpressing cells. Asterisks indicate coexpression of *Rgs4* and *Cadherin16*. (**e**) Cortical detail of an AN mouse kidney showing RNAscope™ signals for *Rgs4* (magenta) and *Havcr1* mRNA (green). Arrows highlight *Rgs4*/*Havcr1*-coexpressing cells. Asterisks highlight *Rgs4*^+^ tubular cells without *Havcr1* expression. (**f**/**g**) Cortical details of AN and UUO kidneys showing RNAscope™ signals for *Rgs5* (magenta) and *Pdgfrb* mRNA (yellow). Asterisks indicate expression of *Rgs5* in tubular structures. (**h**-**i**) Details of kidney sections from control, AN and UUO mice showing RNAscope™ signals for *Rgs16* (magenta), *Adgre-1* (cyan) and *Pdgfrb* mRNA (yellow). Arrows highlight *Rgs16*/*Pdgfrb*-coexpressing cells. In all images nuclei were counterstained with DAPI (grey). Scale bars: 50 µm

In both disease models, *Ado* and *Rgs5* transcript levels were unchanged compared with healthy control kidneys, whereas *Rgs4* and *Rgs16* mRNA levels were significantly increased when analyzed by RT-qPCR (Fig. 5a). While RNAscope™ analyses confirmed the absence of detectable changes in *Ado* expression in kidney sections from the AN model (Supplemental Fig. 2), there were changes in the *Rgs5* expression pattern. *Rgs5* mRNA was clearly detectable in some tubular segments in both fibrosis models (Fig. 5f/g), and—as observed under anemic and PHDi-treated conditions—was also elevated along the *vasa recta*. The number of *Pdgfrb*⁺ interstitial fibroblasts expressing *Rgs5* was only modestly increased in the outer medulla in both AN and UUO relative to healthy controls.

Changes were also evident for *Rgs4*. Compared to healthy controls, kidney sections from both the AN and UUO models exhibited a higher number of *Rgs4*‑positive *Pdgfrb*⁺ interstitial fibroblasts, most prominently in fibrotic lesions of the cortex and the outer stripe of the outer medulla. Additionally, *de novo Rgs4* expression was observed in a subset of *Cdh16*⁺ tubular epithelial cells (Fig. 5b-c).

To further delineate the tubular segments exhibiting *Rgs4* or *Rgs5* expression, we performed segment-specific analysis using established markers: megalin (*Lrp2*) for proximal tubules, Clcnka (chloride voltage-gated channel Ka) for the thin ascending limb of the loop of Henle, NKCC2 (Na–K-2Cl cotransporter, *Slc12a1*) for the thick ascending limb, calbindin (*Calb1*) for distal tubules, and aquaporin 2 (*Aqp2*) for collecting ducts. In addition, we investigated potential co-expression of *Rgs4* and *Rgs5* with markers of tubular injury. These included the proximal tubular injury marker Kim1 (*Havcr1*) [16, 55] and lipocalin-2 (*Lcn2*), which is predominantly upregulated in injured distal tubular segments [31, 37].

In the AN model, tubules expressing *Rgs4* or *Rgs5* were predominantly localized within fibrotic regions. Both isoforms could be detected in *Lrp2*-expressing proximal tubules (Supplemental Fig. 4a). Notably, co-expression of *Lrp2* with *Rgs4* and/or *Rgs5* was frequently observed in areas exhibiting reduced *Lrp2* expression, indicative of early or ongoing proximal tubular injury. Consistent with this, co-expression of both Rgs isoforms with the injury marker *Havcr1* was identified in proximal tubules (Fig. 5e, Supplemental Fig. 4f).

In some cases, both *Rgs4* and *Rgs5* transcripts were upregulated within the same tubular cell, whereas in others only one isoform was induced (Supplemental Fig. 4a). Importantly, *Rgs4* expression was not detected in tubular segments positive for *Clcnka*, *Slc12a1*, *Calb1*, or *Aqp2*. Furthermore, *Rgs4* did not co-localize with the distal injury marker *Lcn2*.

In contrast, sporadic *Rgs5* expression could be detected in individual *Lcn2*-negative cells within *Lcn2*-positive tubular segments (Supplemental Fig. 4h_2_). *Calb1* expression in these segments suggested that they may represent intercalated cells. Most *Lcn2*-expressing tubules, however, were negative for *Rgs5* transcripts (Supplemental Fig. 4h_1_).

In the UUO model, only *Rgs4* mRNA – but not *Rgs5* – was detectable in *Lrp2*- or *Havcr1-*positive proximal tubules, predominantly within regions displaying interstitial fibrosis. Compared with the AN model, fewer injured proximal tubules per kidney section were detected in UUO, which was also reflected by lower co-localization of *Rgs4* with *Havcr1*/*Lrp2*. As in the AN model, all other tubular segments were negative for *Rgs4* expression.

In contrast, induction of *Rgs5* mRNA in intercalated cells of *Calb1*- and *Aqp2*-expressing distal tubular segments was more pronounced than in the AN model. Notably, these segments were frequently positive for *Lcn2*.

Fibrotic lesions are associated with local hypoxia [50]. Notably, induction of *Rgs4* in interstitial fibroblasts and proximal tubular cells was also predominantly observed within fibrotic regions. We therefore analyzed the expression patterns of two well-established HIF-regulated genes, PHD3 (*Egln3*) and Pannexin1 (*Panx1*) [39, 46], in kidney sections from control and AN mice. Hypoxia-induced *Egln3* upregulation has been reported in distal tubular segments, but not in proximal tubules [39]. Furthermore, in our earlier work, we demonstrated that *Egln3* is upregulated in some tubular segments following pharmacological PHD inhibition, but not in interstitial fibroblasts [10]. In healthy kidneys, *Egln3* expression was detected in some tubular segments, interstitial fibroblasts, endothelial cells, vascular smooth muscle cells, and some intraglomerular cells (Supplemental Fig. 5a) [10, 11, 41]. Indeed, AN kidneys exhibited a marked upregulation of *Egln3* in specific tubular segments within fibrotic lesions, as indicated by strong interstitial *Col1a1* mRNA expression (Supplemental Fig. 5b). Within these same fibrotic areas, *Rgs4*-positive tubular segments and interstitial fibroblasts were also present; however, no direct co-localization with *Egln3* transcripts was observed.

Furthermore, a recent study demonstrated HIF-dependent upregulation of *PANX1* mRNA in human primary tubular cells, including both proximal and distal segments [46]. In healthy mouse kidneys, very low *Panx1* expression was detected in *Rgs4*-positive vascular cells and in tubular epithelial cells (Supplemental Fig. 5c) [53]. In AN kidneys, *Panx1* expression was induced within fibrotic lesions, particularly in tubular epithelial cells and a subset of *Col1a1*-positive interstitial cells. Notably, co-expression of *Rgs4* and *Panx1* was observed in some tubular epithelial cells (Supplemental Fig. 5d) as well as in interstitial cells.

The marked induction of *Rgs16* expression was reflected in a substantial increase in *Rgs16*/*Pdgfrb* co-expressing interstitial cells in both models. In the UUO model, these cells were distributed throughout the cortex and medulla, with the highest density and strongest per-cell expression in the inner stripe of the outer medulla – the region exhibiting the most extensive fibrosis. In the adenine model, *Rgs16*/*Pdgfrb* co-expressing cells were primarily localized to the outer medulla and papilla, with cluster-like accumulations also occurring in cortical areas with fibrotic lesions (Fig. 5h-j).

As some macrophages also express *Pdgfrb* mRNA, we examined coexpression of the *Rgs* isoforms with the macrophage marker F4/80 (*Adgre-1*). However, no *Rgs/Adgre-1* coexpression was detectable.

To provide an overview of the changes in renal *Rgs4*, *Rgs5*, and *Rgs16* mRNA expression across the different experimental conditions, the RNAscope™ results are summarized in Table 4.

**Table 4:** Changes in renal *Rgs4*, *Rgs5* and *Rgs16* mRNA expression across cell types and experimental conditions in mice

	VSMCs *Myh11*^+^	Vasa recta		Interstitial cells *Pdgfrb*^+^				Tubular epithelial cells
		Contractile pericytes *Pdgfrb*^+^*/Myh11*^+^	Endothelial cells *Pecam1*^+^	Cortical	Outer stripe outer medulla	Inner stripe outer medulla	Inner medulla	
*Rgs4* mRNA								
Normoxia	+	+ +	/	(+)	(+)	(+)	(+)	/
Anemia	+	+ +	/	+ +	+ +	(+)	(+)	/
PHDi	+	+ +	/	+ + +	+ + +	+ +	+ (+)	/
AN	+	+ +	/	+ + ^1^	+ + ^1^	(+)	(+)	+ + ^2^
UUO	+	+ +	/	+ + ^1^	+ + ^1^	(+)	(+)	+ ^2^
*Rgs5* mRNA								
Normoxia	+ + + +	+ +	+	(+)	+	+	+	/
Anemia	+ + + +	+ + +	+ +	+	+ +	+ +	+	+ ^3^
PHDi	+ + + +	+ + +	+ +	+	+ +	+ +	+	+ ^3^
AN	+ + + +	+ + +	+ +	(+)	+ (+)	+ (+)	+	+ + ^2, (3)^
UUO	+ + + +	+ + +	+ +	(+)	+ (+)	+ (+)	+	+ ^3^
*Rgs16* mRNA								
Normoxia	/	/	/	(+)	(+)	(+)	/	/
Anemia	/	+	/	(+)	(+)	(+)	/	/
PHDi	/	+	/	+	+	+	(+)	/
AN	/	+	/	+ ^1^	+ + ^1^	+ + ^1^	+	/
UUO	/	+	/	+ ^1^	+ + ^1^	+ + ^1^	+	/

/: not detectable, (+): present, but weak signal per cell and few positive cells, + : clearly visible signal per cell, but few positive cells, + + : clearly visible signal per cell, with a moderate number of positive cells, + + + : clearly visible signal per cell, with a high number of positive cells, + + + + : very strong signal per cell, in every renal VSMC, ^1^: within fibrotic lesions, ^2^: proximal tubular segments within fibrotic lesions, ^3^: intercalated cells in the late distal tubule or collecting duct.

#### Localization of ADO-RGS-signaling pathway components in human kidneys

To determine whether the expression patterns of *Ado* and *Rgs* genes observed in rodents are conserved in humans, we analyzed their expression in human kidney biopsy specimens. We examined specimens from 0-biopsies, which experienced a transient undersupply of oxygen due to the lack of blood supply after removal from the donor and thus develop acute tubular damage (mild and severe). In addition, biopsies from patients with mild and progressed IgA nephropathy were used suggesting reduced oxygen supply due to fibrotic changes in glomeruli and interstitium. All analyzed biopsies contained cortical tissue along with variable portions of the medulla.

**Fig. 6:**
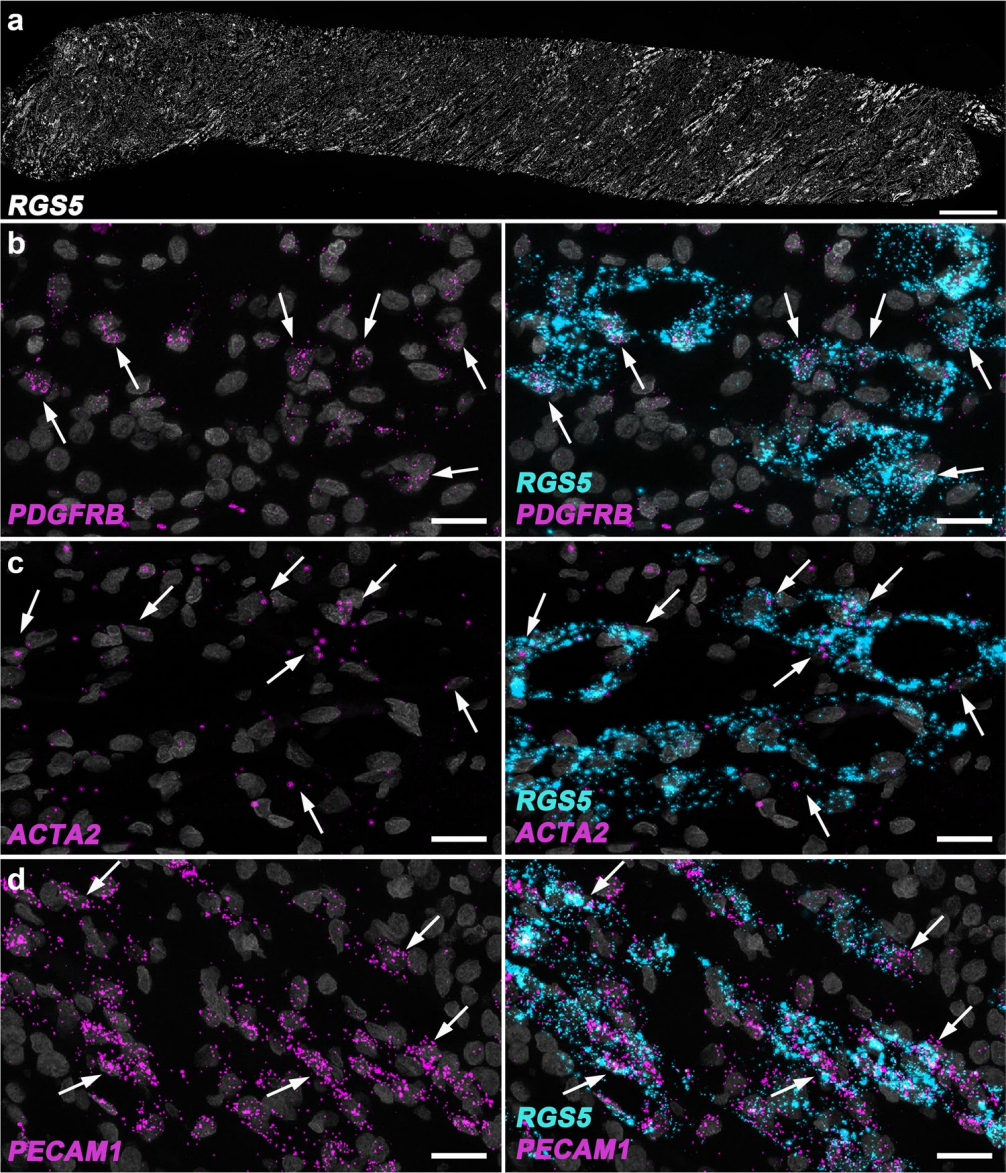
Expression pattern of *RGS5* mRNA in human transplant biopsies with acute tubular injury. (**a**) Overview showing the distribution of *RGS5* mRNA (white) detected by RNAscope™. Scale bar: 500 µm. (**b**) Medullary detail showing only *PDGFRB* mRNA (magenta, left) or co-expression with *RGS5* (cyan, right) detected by RNAscope™ along the *vasa recta*. Arrows highlight double positive cells. Scale bars: 20 µm. (**c**) Medullary detail showing only *ACTA2* mRNA (magenta, left) or co-expression with *RGS5* (cyan, right) detected by RNAscope™. Arrows highlight double positive cells. Scale bars: 20 µm. (**d**) Medullary detail showing only *PECAM1* mRNA (magenta, left) or co-expression with *RGS5* (cyan, right) detected by RNAscope™. Arrows highlight double positive cells. Scale bars: 20 µm. In all images nuclei are counterstained with DAPI (grey)

No *ADO* or *RGS16* transcripts could be detected in any of the human specimens analyzed. In contrast, *RGS5* mRNA expression was clearly present in small vascular structures of all analyzed biopsies, regardless of whether the tissue exhibited mild or severe injury. Co-expression of *PDGFRB* and *ACTA2* (α-smooth muscle actin, α-SMA) in these *RGS5*-positive cells identified a subset of them as pericytes, while co‑expression of *PECAM1* indicated that some *RGS5*‑expressing cells were endothelial cells (Fig. 6). These findings were consistent with our observations along the *vasa recta* in mice. Additionally, vascular smooth muscle cells of larger vessels as well as afferent and efferent arterioles also displayed *RGS5* mRNA expression. Unlike in mice, there was no obvious difference in the *RGS5* mRNA expression level per cell between VSMCs of larger arteries and afferent/efferent arterioles or pericytes of the *vasa recta*. Moreover, we could not detect any differences in the *RGS5* expression levels per cell along the *vasa recta* between mild or severe/progressed injury conditions, while in mice *Rgs5* expression was upregulated following hypoxia or experimental fibrosis. Moreover, *RGS5* mRNA expression was not observed in tubular epithelial cells or medullary interstitial fibroblasts in biopsies from patients with either acute tubular injury or IgA nephropathy.

**Fig. 7:**
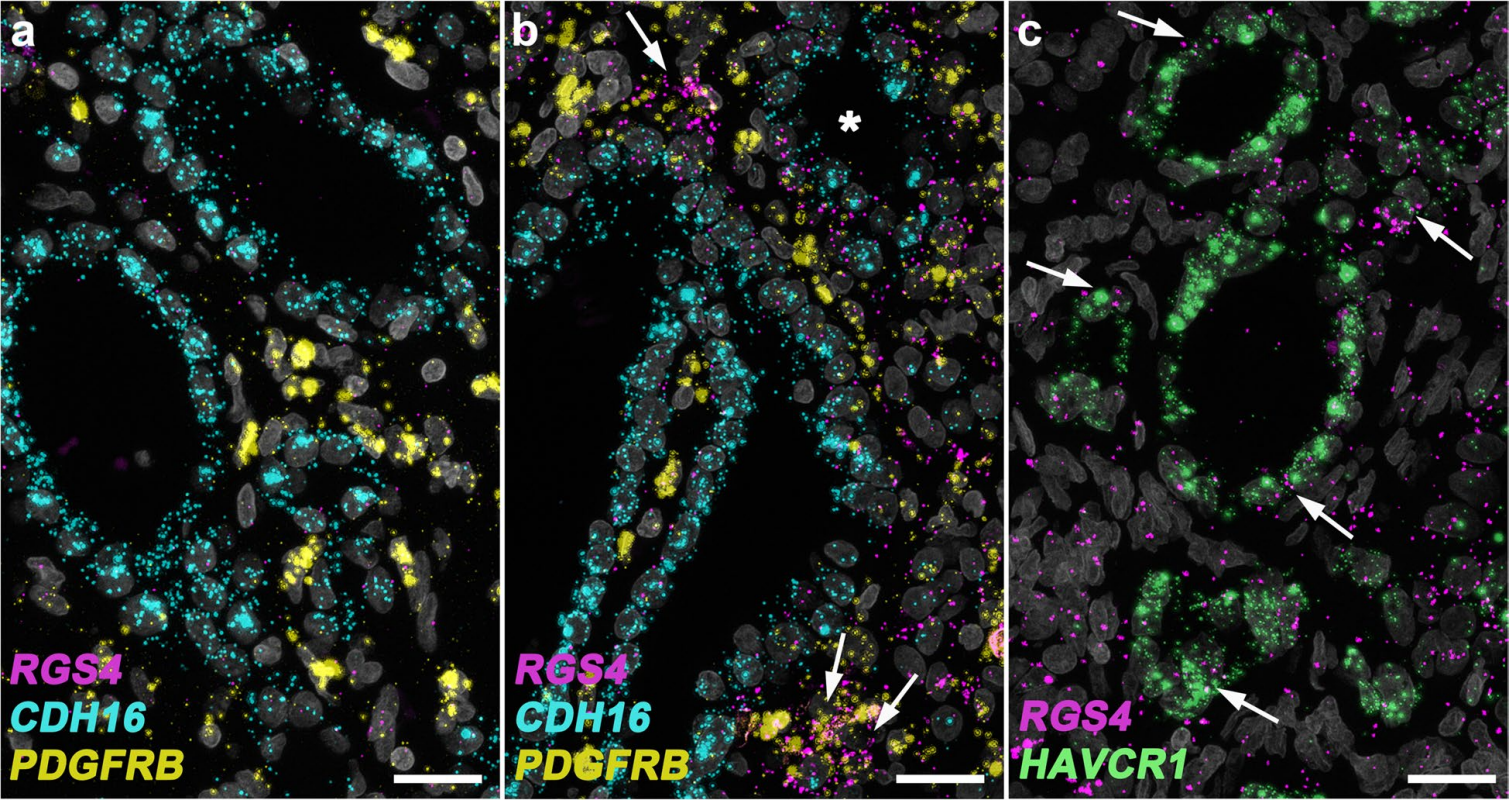
Localization of *RGS4* mRNA in human biopsies of patients with mild and progressed IgA nephropathy. (**a**/**b**) Cortical details displaying triple RNAscope™ for *RGS4* (magenta), *CDH16* (cyan), and *PDGFRB* (yellow) in patients without global glomerulosclerosis (**a**) and more than 70% of glomeruli showing global glomerulosclerosis (**b**). Arrows highlight cells co‑expressing *RGS4* and *PDGFRB* mRNA, while the asterisk indicates a tubular segment positive for both *RGS4* and *CDH16* mRNA. (**c**) Cortical detail showing RNAscope™ signals for *RGS4* (magenta) and *HAVCR1* mRNA (green) in a patient with 75% of glomeruli with global glomerulosclerosis. Arrows indicate *RGS4*/*HAVCR1* co-expression. In all images nuclei are counterstained with DAPI (grey). Scale bars: 20 µm

As observed in fibrotic murine kidneys, *RGS4* mRNA was detected in cortical and corticomedullary regions exhibiting interstitial fibrosis in both patients with progressed IgA nephropathy, where it co-localized with *PDGFRB*. *RGS4* expression was also observed in some *CDH16*-positive tubular epithelial cells within these fibrotic lesions. In some cases, co-expression of *RGS4* with the proximal tubular injury marker *HAVCR1* was detected (Fig. 7). In contrast, the biopsy from the patient with only mild IgA nephropathy showed no *RGS4* expression in either tubular or interstitial cells in the cortex or medulla (Fig. 7a). A similar pattern was observed in biopsies showing acute tubular injury: *RGS4* mRNA signals were present in injured tubular cells or interstitial cells in the cortex and corticomedullary region in patients with severe injury but absent in the patient with mild damage.

In contrast to murine kidneys, *RGS4* mRNA expression was only detected in VSMCs but not in pericytes along the *vasa recta* in human biopsies.

## Discussion

Oxygen‑dependent enzymes act as hypoxia sensors and mediate cellular adaptation to reduced oxygen levels. While many responses are governed by the classical HIF pathway [29, 40], additional oxygen‑dependent enzymes have recently been identified, including the cysteamine dioxygenase ADO [22, 32]. ADO modifies specific RGS proteins in an oxygen‑dependent manner, targeting them for proteasomal degradation via the N‑degron pathway. Under hypoxia, these RGS isoforms evade degradation and function as negative regulators of G‑protein signaling [18]. Given the kidney’s high sensitivity to hypoxia, the oxygen‑dependent ADO–RGS pathway may be involved in renal cellular adaptation when oxygen availability declines.

For this reason, our study aimed to identify the renal cell types in which the ADO–RGS pathway may operate by localizing *Ado* and *Rgs4*, *Rgs5*, and *Rgs16* gene expression in mice and humans under normoxic, hypoxemic, and pathophysiological conditions.

Our spatial analyses of mouse kidney sections revealed clear *Ado* mRNA expression across all examined cell types, including tubular epithelial cells, endothelial cells, VSMCs, contractile pericytes, and interstitial fibroblasts. Although expression levels per cell were uniformly low, more than 90% of cells within each population were positive for *Ado* mRNA (Fig. 1). In contrast, no reliable *ADO* signals were detected in human biopsy specimens. The RNAscope™ findings in mice are consistent with the cellular distribution of *Ado/ADO* expression reported in single-cell transcriptomic datasets from healthy mouse kidneys [38], the Human Protein Atlas (https://www.proteinatlas.org/ENSG00000181915-ADO/single+cell; available from v25.0.proteinatlas.org) and the Kidney Precision Medicine Project (KPMP; https://atlas.kpmp.org) [9, 23, 44], but suggest that a lot more cells express *Ado* than reported with RNAseq. In these datasets fewer than 10% of cells per cell type in the mouse express *Ado*, and the corresponding reads in human samples are so underrepresented that they fall below thresholds considered statistically significant. Based on these reports and considering that per-cell *Ado* expression was also low in optimally preserved mouse tissue, it is likely that RNAscope™ lacked the sensitivity required to detect *ADO* transcripts in archived human kidney specimens, which were primarily processed for routine histopathological evaluation. Therefore, the absence of detectable *ADO* signals in human tissue most likely reflects technical limitations rather than true absence of gene expression.

Across all experimental conditions, *Ado* mRNA expression remained remarkably stable. Neither anemia, PHD inhibition, nor the pathophysiological models examined resulted in altered expression patterns or overall renal transcript levels in mice. These findings are consistent with a previous report demonstrating that hypoxia does not influence *ADO* mRNA expression in a neuroblastoma cell line [49]. The ubiquitous and condition‑independent expression of *Ado* suggests that oxygen‑dependent regulation of Ado-targeted *Rgs* isoforms is, in principle, possible in any renal cell type and may depend primarily on the expression profile of the respective Rgs isoforms.

Indeed, our spatial analysis of the Rgs isoforms revealed clearly distinct expression patterns and specific changes under the analyzed conditions. *Rgs16* expression was localized exclusively to *Pdgfrb*^+^ interstitial cells. While only very few *Rgs16*^+^ cells were detectable in the healthy normoxic kidney, pharmacological HIF stabilization induced *Rgs16* transcription predominantly in some interstitial *Pdgfrb*^+^ fibroblasts of the outer medulla (Fig. 3, Fig. 4). These changes were even more pronounced in both kidney fibrosis models, where *Rgs16* mRNA expression was strongly upregulated in a substantial subset of *Pdgfrb*^+^ interstitial cells, particularly within fibrotic regions. Previously, *Rgs16* expression has been described in immune cells [42, 47] suggesting a potential role in modulating inflammatory responses [48]. Of note, although we were not able to detect *RGS16* transcripts in human biopsies, KPMP transcriptomic data confirm *RGS16* expression in fibroblasts [9], consistent with our observations in the mouse.

The predominant localization of *Rgs4* and *Rgs5* in VSMCs of renal arteries and arterioles, as well as in contractile medullary pericytes along the *vasa recta* in healthy normoxic kidneys aligns well with previous studies reporting *Rgs4* or *Rgs5* expression in vascular cells in the kidney and other organs [1, 3, 13, 26, 43, 52]. This is also consistent with *Rgs5* being an organ‑independent marker for pericytes [3]. In agreement with our earlier work, the small number of *Rgs4*-expressing interstitial fibroblasts in the cortex corresponded to Epo-producing cells, whereas the majority of interstitial fibroblasts in both cortex and medulla lacked detectable *Rgs4* signals [4]. In contrast, the few *Rgs5-* expressing interstitial fibroblasts under normoxic conditions were predominantly located in the medullary region and not in the cortex. Importantly, this zonal separation of *Rgs4* and *Rgs5* expression in interstitial *Pdgfrb*^+^ fibroblasts was largely preserved during the induction of both isoforms under anemia and across both pathophysiological models. *Rgs4* induction was observed mainly in cortical fibroblasts, whereas *Rgs5* induction occurred preferentially in medullary fibroblasts. An exception was observed for *Rgs4* induction in interstitial fibroblasts following PHD inhibition, where induction extended to fibroblasts across all kidney zones (Fig. 3b). This likely reflects the markedly greater extent of HIF-2α stabilization after PHDi treatment compared with anemia. In line with our previous findings, PHDi induced HIF-2α stabilization in interstitial fibroblasts throughout all renal zones, whereas under anemic conditions, HIF-2α–positive fibroblasts were restricted to the cortex and the outer stripe of the outer medulla (Supplemental Fig. 3) [10]. In contrast, the *Rgs5* induction pattern in interstitial fibroblasts did not differ between anemia and PHDi conditions. Notably, fibroblasts in the outer stripe of the outer medulla upregulated both isoforms (Fig. 3, Fig. 4). This differential distribution of *Rgs4* and *Rgs5* expression in renal fibroblasts may result from distinct *Rgs* expression profiles characteristic of the different subtypes of renal PDGFR-β⁺ interstitial cells [4].

Importantly, both chronic kidney fibrosis models – AN and UUO – revealed *Rgs4* and *Rgs5* mRNA induction patterns similar to those observed under acute hypoxemic conditions: *Rgs4* expression was enriched in interstitial *Pdgfrb*⁺ fibroblasts in the cortex and the outer stripe of the outer medulla, corresponding to fibrotic regions, while *Rgs5* transcription was upregulated in some medullary fibroblasts and along *vasa recta*. Notably, in the AN model we also observed transcription of both *Rgs* isoforms in (injured) proximal tubular epithelial cells within fibrotic lesions, as indicated by the coexpression of *Lrp2* (megalin) and the proximal tubular injury marker *Havcr1 (*Kim-1*)* (Fig. 5, Supplemental Fig. 4). Sporadically, *Rgs5* transcripts were detected in intercalated cells of injured *Lcn2*^+^ distal tubular segments, as was observed after PHD inhibition (Supplemental Fig. 1 and 4). The predominant induction of *Rgs* isoforms in fibrotic areas is consistent with the concept that extensive extracellular matrix deposition creates localized (chronic) hypoxic niches [50], which may promote induction of *Rgs4* and *Rgs5* in these regions. This is further supported by the induction of the HIF-target genes *Egln3* and *Panx1* within the same fibrotic areas (Supplemental Fig. 5) [10, 39, 46]. However, because *Rgs4* transcripts were not detectable in tubular epithelial cells under anemia or PHDi alone, additional *Rgs*-inducing factors are likely involved in the context of fibrosis. Indeed, the profibrotic mediators angiotensin II and interleukin-1β have been reported to induce *Rgs4* transcription [20]. Of note, in the UUO model, only *Rgs4* expression was induced in proximal tubular segments, whereas – compared to AN – *Rgs5* induction was more pronounced in intercalated cells of injured distal tubular segments. Whether the observed differences in the extent of proximal tubular injury and/or potential differences in the spatial distribution of fibrotic lesions (and thus hypoxic regions) contribute to the observed divergence in *Rgs5* induction between the two models needs to be further investigated in future experiments.

Consistent with the murine data, *RGS5* showed robust expression in both large and small vessels in the cortex, as well as in *PDGFRB/ACTA2*⁺ pericytes and *PECAM1*⁺ endothelial cells within vascular bundles of the outer medulla across all human kidney specimens. However, unlike in mice, neither acute nor chronic hypoxia appeared to alter *RGS5* mRNA expression along the *vasa recta*, nor did it induce *RGS5* expression in interstitial fibroblasts or tubular epithelial cells. In contrast, *RGS4* expression correlated with the severity of both acute and chronic injury. It was detectable in interstitial *PDGFRB*⁺ fibroblasts and in *HAVCR1*⁺ injured proximal tubular segments in 0-biopsies of transplants with severe tubular damage and in progressive IgA nephropathy but remained absent in cases of mild injury—mirroring the induction pattern observed in mouse kidneys under pathophysiologic conditions (Fig. 6, Fig. 7). However, in contrast to murine kidneys, *RGS4* mRNA expression was not detected in pericytes along the *vasa recta* but only in VSMCs. Moreover, whether *RGS4* in healthy human kidneys would exhibit a specific zonal distribution pattern or co-induction with EPO under hypoxic conditions or after PHD inhibition, as observed in murine tissue, could not be determined based on the available biopsies.

Although our findings suggest potential species-specific differences in *RGS* expression in certain cell types, the overall results nevertheless provide compelling evidence that functional insights into RGS4 (and RGS5) obtained from cell-type-specific knockout mouse models may have translational relevance. This is particularly noteworthy for RGS4, as no appreciable *RGS4* expression has been reported in human kidney cells within the KPMP RNA-seq datasets to date [9].

The observation that both anemia and the pharmacological stabilization of HIF‑α by PHD inhibitors (under otherwise normoxic oxygen availability) each resulted in similar expression patterns of *Rgs4* and *Rgs5* suggests that transcriptional regulation of these genes includes a HIF‑dependent component. The induction of *Rgs16* in medullary fibroblasts and pericytes following PHDi treatment further supports a HIF‑mediated regulatory mechanism. Consistent with the present findings, we previously observed a HIF‑2α‑dependent induction of *Rgs4* expression in cortical and corticomedullary fibroblasts not only under anemic conditions but also following low‑oxygen exposure and CO inhalation [4].

However, the transcription of the analyzed *Rgs* isoforms does not appear to be universally HIF-regulated across all cell types or species. This is illustrated by the unchanged *Rgs4* and *Rgs5* expression levels in mouse VSMCs under all tested conditions, as well as by the lack of *RGS5* upregulation in human kidneys with acute or chronic hypoxic injury. In contrast, along the *vasa recta*, *Rgs5* transcription was clearly induced in both pericytes and endothelial cells in mice (Fig. 3, Supplemental Fig. 1). These seemingly contradictory findings align with previous reports. For instance, while a HIF‑2α‑dependent increase in *RGS4* expression has been shown in several tumor cell lines, no such induction was observed in vascular cell lines [35]. Conversely, a HIF‑dependent rise in *RGS5* mRNA has been described in human umbilical vein endothelial cells [19], whereas cultured human brain pericytes showed unchanged *RGS5* mRNA levels over 24 h of hypoxia exposure [8]. These observations indicate that potential HIF-dependent regulation must be carefully evaluated for each individual cell type. Supporting this notion, no HIF-binding site could be identified within 32 kb of the human *RGS4* gene locus, suggesting that HIF-dependent regulation of RGS isoforms may be mediated through long-distance genomic interactions [35].

Physiologically, a HIF-dependent regulation of the analyzed *Rgs* isoforms suggests an interaction between the two oxygen‑sensitive signaling pathways – the HIF pathway and the ADO-RGS axis – in the adaptation of specific cell types to hypoxemia. One may speculate that the HIF-dependent transcriptional upregulation of specific RGS isoforms results in an increased number of stabilized RGS proteins (as they are not oxygenated by ADO due to hypoxia and therefore escape degradation), which could in turn modulate GPCR signaling pathways. Such modulation may either reinforce or counteract other HIF-mediated adaptive responses to hypoxia, for example metabolic adaptations, given that both HIF signaling and GPCR signaling participate in metabolic regulation [33, 56].

As a role for RGS5 in regulating vascular tone [30], vascular remodeling [28] and preserving endothelial cell function [27] has been established, the robust *Rgs5* upregulation along the *vasa recta* in both hypoxemia and fibrotic kidney disease models may indicate a role in regulating and ensuring medullary blood flow in response to a decline in oxygen availability.

RGS4, by contrast, may have a more pro‑fibrotic function, as has been reported in cardiac fibrosis. In the heart, *Rgs4* was shown to modulate both the TGF‑β/Smad and MAPK signaling pathways, which regulate processes such as proliferation, differentiation, and apoptosis [14]. An influence of RGS4 on MAPK signaling has also been described in the context of tubulogenesis during kidney development [2]. RGS5, on the other hand, exhibited predominantly anti‑fibrotic effects in the heart [25]. Thus, stabilization of both isoforms by ADO in the same cell could potentially balance pro‑ and anti‑fibrotic effects, whereas activation of only one isoform may shift the response toward the corresponding pro‑ or anti‑fibrotic function.

Another question concerns the functions of RGS4 and RGS5 in interstitial fibroblasts under hypoxemic conditions. Notably, *Epo* and *Rgs4* exhibit consistent co‑expression. This raises the possibility of a feedback effect of RGS4 on EPO synthesis, since both *Epo* and *Rgs4* transcription are upregulated in a HIF‑2‑dependent manner during hypoxia. The signaling pathways that might mediate such regulation remain unclear and require further investigation. One conceivable mechanism is activation of the TGF‑β1 signaling pathway by RGS4, as described in the heart [14]. Supporting this notion, we previously showed that fibroblast‑specific deletion of the TGF‑β receptor 2 led to increased EPO production under normoxic conditions and preserved *Epo* expression during fibrosis [12]. Cell type–specific functional analyses will be necessary to define the renal roles of RGS4 and RGS5 within the context of ADO–RGS signaling under hypoxemic and fibrotic conditions.

We are fully aware that the analyses presented in this study are limited to mRNA expression data, and that (due to so far unsuccessful attempts to establish reliable immunofluorescence staining protocols) we cannot yet state with certainty that the observed transcriptional upregulation in specific cell types necessarily results in protein translation and subsequent stabilization of the corresponding RGS proteins. However, the primary aim of this study was to identify the relevant cell types and spatial expression changes in order to determine in which cells the ADO–RGS signaling pathway might play a role to provide a basis for subsequent functional studies using cell-type specific knockout mouse models. Moreover, previous reports indicate that the activity and cell type-specific functions of RGS proteins can indeed be regulated at the transcriptional level [45, 54], further underscoring the relevance of our findings.

Overall, this study provides a systematic and spatially resolved characterization of renal expression patterns of the O_2_‑sensing ADO–RGS axis and their alterations in response to hypoxia or kidney disease. We identified previously unrecognized *Rgs*‑expressing cell types in both murine and human tissue, as well as distinct changes in the expression of *Rgs4*, *Rgs5*, and *Rgs16* – particularly within *Pdgfrb⁺* interstitial cells and tubular epithelial cells – while *Ado* displayed consistently robust expression across conditions. Together, these findings establish an important basis for future studies aimed at elucidating cell type-specific functions of ADO-RGS signaling in the kidney.

## Supplementary Information

Below is the link to the electronic supplementary material.

Supplementary file1 (DOCX 320 MB)

## Data Availability

Original datasets are available from the corresponding author upon reasonable request.
