# Supplementary material for: Localization of O₂‑sensing ADO‑RGS pathway components in mouse and human kidneys under normoxia, hypoxia and renal fibrosis
Source: Pflugers Arch. 2026 Jun 25;478(7):59. doi: 10.1007/s00424-026-03188-7 (PMC13294159; doi:10.1007/s00424-026-03188-7)
Supplement: Supplementary file 1 — Supplementary file1 (DOCX 320 MB) [file 424_2026_3188_MOESM1_ESM.docx]

**Supplemental Figures:**

**Supplemental Fig. 1** Expression patterns of *Rgs5* mRNA in normoxic, anemic and PHDi-treated mice

(a-c) Medullary details showing the coexpression of *Rgs5* (magenta) with *Pdgfrb* (yellow), or *Pecam1* mRNA (cyan) along *vasa recta* detected by RNAscope^TM^ under normoxic, anemic and PHDi-treated conditions. Arrows highlight *Rgs5* positive endothelial (*Pecam1*) cells. (d) Detail of a collecting duct from a PHDi-treated mouse showing an RNAscope^TM^ for *Rgs5* (magenta) and *Aqp2* mRNA (cyan).

In all images nuclei are counterstained with DAPI (grey). Scale bars: 30 µm.

**Supplemental Fig. 2** Renal expression patterns of *Ado* mRNA in normoxic, anemic, PHDi-treated and AN mice

(a) Details from kidney sections under normoxic, anemic, PHDi-treated and AN conditions showing the zonal distribution of *Ado* (red) mRNA expression using RNAscope^TM^. Dotted white lines indicate the zonal borders. Circles indicate glomeruli. Nuclei are counterstained with DAPI (grey). Scale bars: 500 µm. (b) Automated image analysis shows the *Ado* positive area per indicated kidney zone relative to the nuclei area under the respective conditions. Data represent mean ± SD of n ≥ 4 mice per condition.

**Supplemental Fig. 3** HIF-2α protein stabilization on kidney sections under normoxic, anemic and PHDi-treated conditions

HIF‑2 stabilization was assessed by immunohistochemical staining for HIF‑2α, visible as a brown nuclear signal, in kidney sections of wildtype mice under normoxic (top panel), anemic (left column) and PHDi-treated conditions (right column). Under normoxic conditions, a representative region including the cortex and the outer stripe of the outer medulla is shown. Only very few HIF‑2α positive interstitial fibroblasts (arrow) can be detected under normoxic conditions.

For anemia and PHDi treatment, zonal details depict the cortex, outer medulla, and inner medulla, respectively. Under anemia, clusters of HIF‑2α positive fibroblasts could be detected in the cortex along with some HIF‑2α positive fibroblasts in the outer stripe of the outer medulla (arrows), but none in the inner medulla. After PHDi treatment most interstitial fibroblasts across all kidney zones were positive for HIF-2α. Moreover, HIF-2α was stabilized in intraglomerular mesangial cells, endothelial cells and vascular smooth muscle cells. Circles indicate glomeruli. Scale bars: 50µm.

**Supplemental Fig. 4** Co-expression of *Rgs4* and *Rgs5* transcripts with specific tubular markers and injury markers on kidney sections of mice with adenine-induced nephropathy

(a-e) Details of kidney sections from AN mice showing triple RNAscope^TM^ for *Rgs4* (magenta), *Rgs5* (cyan) and the proximal tubular marker *Lrp2* mRNA (yellow, a), *Clcnka* (chloride voltage-gated channel Ka) for the thin ascending limb of the loop of Henle (b), NKCC2 (*Slc12a1*) for the thick ascending limb (c), calbindin (*Calb1*) for distal tubules (d), and *Aqp2* for collecting ducts (e). Arrows highlight some *Rgs4*/*Rgs5*/*Lrp2* co-expressing cells. (f) Cortical detail showing RNAscope^TM^ signals for *Rgs5* (magenta) and *Havcr1* mRNA (green). Arrows highlight *Rgs5*/*Havcr1* co-expressing cells. (g) Detail showing RNAscope^TM^ signals for *Rgs4* (magenta) and the injury marker *Lcn2* (green). (h) Details showing an RNAscope^TM^ for *Rgs5* (magenta) and *Lcn2* mRNA (green). Sporadically *Rgs5* expression could be detected in individual cells (arrows) within *Lcn2*-positive tubular segments (h_2_).

In all images nuclei are counterstained with DAPI (grey). Scale bars: 50 µm.

**Supplemental Fig. 5** Cohybridization of *Rgs4* transcripts with the HIF-target genes *Egln3* or *Panx1* and the interstitial fibrosis marker *Col1a1* on kidney sections of control mice and mice with adenine-induced nephropathy

(a/b) Cortical details showing RNAscope™ for *Egln3* (magenta), *Rgs4* (cyan) and *Col1a1* (yellow) mRNA on kidney sections from control (a) and AN mice (b). In control kidneys, *Egln3* was expressed in some tubular segments, interstitial fibroblasts, endothelial cells, vascular smooth muscle cells, and intraglomerular cells. In AN kidneys, *Egln3* was upregulated in specific tubular segments within *Col1a1*-positive fibrotic lesions. *Rgs4*-positive tubular segments were also detected within these same fibrotic regions (b).

(c/d) Cortical details showing RNAscope™ for *Panx1* (magenta), *Rgs4* (cyan) and *Col1a1* (yellow) mRNA on kidney sections from control (c) and AN mice (d). In control kidneys, *Panx1* expression was very low. Weak expression could be detected in *Rgs4*-positive vascular structures as well as in some tubular epithelial cells (c). In AN kidneys, *Panx1* was upregulated in tubular cells and *Col1a1*-positive interstitial cells within fibrotic lesions. Notably, in some tubular segments, coexpression of Rgs*4* and *Panx1* could be observed (d).

In all images nuclei are counterstained with DAPI (grey). Circles indicate glomeruli. Scale bars: 50 µm.
